# Mating Damages the Cuticle of *C. elegans* Hermaphrodites

**DOI:** 10.1371/journal.pone.0104456

**Published:** 2014-08-08

**Authors:** Gavin C. Woodruff, Christine M. Knauss, Timothy K. Maugel, Eric S. Haag

**Affiliations:** 1 Department of Biology, University of Maryland, College Park, Maryland, United States of America; 2 Laboratory for Biological Ultrastructure, University of Maryland, College Park, Maryland, United States of America; 3 Forest Pathology Laboratory, Forestry and Forest Products Research Institute, Tsukuba, Ibaraki, Japan; Centre National de la Recherche Scientique & University of Nice Sophia-Antipolis, France

## Abstract

Lifespan costs to reproduction are common across multiple species, and such costs could potentially arise through a number of mechanisms. In the nematode *Caenorhabditis elegans*, it has been suggested that part of the lifespan cost to hermaphrodites from mating results from physical damage owing to the act of copulation itself. Here, we examine whether mating damages the surface of the hermaphrodite cuticle via scanning electron microscopy. It is found that mated hermaphrodites suffered delamination of cuticle layers surrounding the vulva, and that the incidence of such damage depends on genetic background. Unmated hermaphrodites demonstrated almost no such damage, even when cultured in soil with potentially abrasive particles. Thus, a consequence of mating for *C. elegans* hermaphrodites is physical cuticle damage. These experiments did not assess the consequences of cuticle damage for lifespan, and the biological significance of this damage remains unclear. We further discuss our results within the context of recent studies linking the lifespan cost to mating in *C. elegans* hermaphrodites to male secretions.

## Introduction

Tradeoffs between lifespan and reproduction are thought to play a crucial role in the evolution of life history traits [1]. Such tradeoffs are widespread in metazoans [2]–[4], and have been traditionally framed within a theoretical context of limited organismal energetic resources. That is, it is often presumed that use of energetic resources on lifespan (or the soma) has a detrimental consequence on reproduction (or the germline), and vice-versa [5]–[7]. However, lifespan costs to reproduction are not always limited to such metabolic tradeoffs. For instance, such costs can arise due to increased exposure to predation [8], disease [9], costs to foraging time [10], the inhibition of proper immune function [11], the loss of egg-laying ability [12] and physical damage [13], among others. Thus, lifespan costs to reproduction take many forms and must entail a variety of specific mechanisms across species.

Furthermore, costs to reproduction are expected to be asymmetrical in males and females due to the sex-specific differential investment in gamete size or anisogamy [14]. Anisogamy is presumed to set the stage for the evolution of sexually antagonistic traits [15]. Such sexual conflicts, which arise when traits that are beneficial in one sex harm the other, are now understood to be widespread and have been found in a wide range of taxa [16], including spiders [17], birds [18], and fruit flies [19]. Thus, the study of lifespan costs to reproduction, when asymmetrical in males and females, can also provide insights into evolutionary sexual conflicts.

The nematode *Caenorhabditis elegans* is well-suited to the study of such phenomena. Standardized techniques allow interrogation of a wide variety of hypotheses, and it also exhibits a sex-biased lifespan cost to mating [20]. In *C*. *elegans*, there is a lifespan cost to mating for hermaphrodites, but not for males (when mated with hermaphrodites [20]; lifespan costs do result when males mate with other males [21]). A number of genetic experiments has led to the hypothesis that the mechanism of this cost is due to the damage incurred during copulation itself, and not due to any metabolic tradeoffs between the soma and the germline [20]. On the other hand, recent studies [22], [23] suggest that secreted ascaroside molecules and male seminal fluid are responsible for this cost.

The nature of mating in *Caenorhabditis* suggests that localized surface damage may occur around the vulva. Copulation involves the use of the male tail to scan the hermaphrodite body surface for the vulva [24]. Once the vulva is detected, the male will then use its scleritized spicules to prod the vulva, the spicules are inserted into the vulval slit to pry it open, and then sperm are ejaculated into the uterus [24]. More specifically, before spicule insertion, there is a prolonged period of rapid spicule prodding of the vulva area [25]. Reportedly, this period of prodding is typically greater than ten minutes long, and males prod the area at an average of about seven times per second [25]. It would therefore not be surprising if mating causes hermaphrodites to suffer some sort of physical damage.

Here, the hypothesis that mating incurs physical damage to *C*. *elegans* hermaphrodites is interrogated using scanning electron microscopy (SEM). It is revealed that mating results in delamination of cuticle layers localized to the vulval area in hermaphrodites. Unmated hermaphrodites show almost no such damage. Thus, one of consequences of reproduction in *C*. *elegans* hermaphrodites is cuticle damage, and under some circumstances this may contribute to the corresponding lifespan reduction.

## Methods

### Maintenance and strains

Animals were maintained according to standard *C*. *elegans* procedures [26], with the exception of increasing agar concentration in NGM plates to 2.2% in order to discourage animals from burrowing underneath the surface of the plate. Cultures were raised at 20°C. Strains used in this study include: N2, *him-5* (*e1490*) DR466, and the non-plugging strain QG2288. QG2288 was derived initially from two lines of *C. elegans* (AB2 and CB4856) that are known to have males that are particularly good at copulating (CB4856 males produce more cross-progeny and have more successful copulations than N2 males [27]) or that display exceptionally aggressive sexual behavior (AB2 males frequently deposit copulatory plugs on their own heads and the heads of other males, which N2 males do not do [21]). To generate the non-plugging strain, *him-5(e1490)*-bearing AB2 and CB4856 lines were crossed to generate F2, and then were inbred by selfing for ten generations to create the recombinant inbred line (RIL) QG71. Then, the N2 allele of *plg-1* (which renders males unable to produce a copulatory plug [28]) was introgressed into QG71 to produce the line QG2288 (pers. comm, M.V. Rockman). QG2288 is thus a non-plugging strain that may have a higher male-mating efficiency than N2-derived stains due to its parental genetic background.

### Culture conditions

L4 hermaphrodites were placed onto *E. coli* OP50-seeded NGM plates and either left alone or mated with conspecific males at a ratio of seven hermaphrodites to ten males per plate for five days. Worms were moved every two days to ensure they were not confused with their progeny. Males were also moved with hermaphrodites, but rare dead and missing males were replaced with young males. After five days, hermaphrodites were prepared for SEM.

To compare the stress of mating with the potential stress of the natural environment, a separate series of observations were performed on dirt plates. Topsoil from the University of Maryland, College Park campus was washed with distilled water several times to remove soluble materials, boiled for 30 min. to kill fungal spores, drained, and then autoclaved in a glass bottle. Roughly 15 ml of loosely packed, sterilized soil was mixed with 5 ml of a dense overnight culture of OP50 and spread onto unseeded NGM plates (9 cm diameter). After overnight incubation at room temperature to allow bacterial growth, young adult N2 hermaphrodites were added via a platinum picker to the plates at the soil margin. Worms quickly entered the soil and disappeared. After five days at 20°C, hermaphrodites from both dirt and dirt-less plates were washed from the soil with M9 buffer and the largest animals (presumably from the original founding cohort) were prepared for SEM.

### Electron microscopy

Worms were transferred to 0.12 M phosphate buffer and fixed by adding glutaraldehyde to 2% and incubating overnight at room temperature. They were then washed in buffer before post-fixing for 30 minutes in 1% osmium tetroxide in the same buffer. Dehydration in a graded ethanol series preceded drying from liquid carbon dioxide by the critical point method. The samples were then mounted onto stubs and sputter coated with 10 nm of gold palladium alloy before being imaged in an Amray 1820 scanning electron microscope. Scanning electron microscopy images were analyzed for the quantitative extent of vulva damage using the “Measure Area” feature of the ImageJ application [29]. The area of vulva delamination for each image was encircled by a free hand selection, and the resultant area in arbitrary units was converted to square microns.

## Results

**Figure 1:**
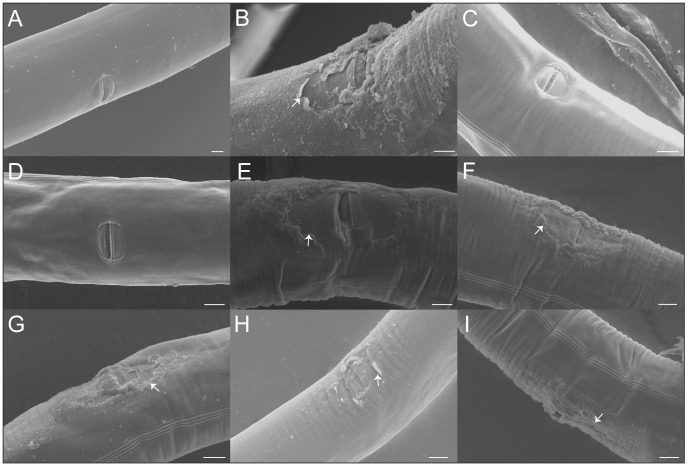
Mating causes vulva cuticle damage in *C*. *elegans* hermaphrodites. (A) The vulva of an unmated N2 hermaphrodite. (B) The vulva of a N2 hermaphrodite mated with a male with an N2 background. The arrow denotes where delamination of the cuticle surface near the vulva has occurred. (C–D) Unmated QG2288 hermaphrodites with no apparent physical damage. (E) A rare unmated QG2288 hermaphrodite with damage near the vulva (arrow). (F–I). QG2288 hermaphrodites mated with males of the same strain. Arrows denote the presence of cuticle tearing near the vulva. Scale bars represent ten microns in all panels.

To evaluate the hypothesis that mating promotes physical damage in hermaphrodites, hermaphrodites either unmated or continuously mated with males for five days were examined under SEM. Initially, this was performed with the N2 strain of *C. elegans*, a standard laboratory strain known to have low male mating efficiency [27], [30]. In the N2 background, unmated hermaphrodites showed no evidence of physical damage (Figure 1A; Table 1). However, a non-significant fraction of mated N2 hermaphrodites displayed tearing and delamination of the cuticle localized to the vulva region (Figure 1B; Table 1).

**Table 1:** Incidence of vulva cuticle damage in mated and unmated hermaphrodites.

♂+ Strain	♂ Strain	Fraction unmated ♂+ with physical damage	Fraction mated ♂+ with physical damage	P-value*
N2	DR466	0/8	5/9	0.0824
QG2288	QG2288	3/18	20/20	<0.0001

Fractions represent the number of animals with vulval cuticle damage out of the total number of animals observed. DR461 is a strain with a *him-5* mutation in an N2 background. ♂+  =  hermaphrodites, ♂  =  males. *Fisher's Exact Test.

Because N2 males are known to be relatively poor maters, we repeated the experiment with another, genetically distinct *C*. *elegans* strain, QG2288 (see Methods). This strain was utilized because it is derived from robustly mating parental strains, yet fails to deposit copulatory plugs, which would presumably obstruct the observation of cuticle damage. In this case, all mated hermaphrodites displayed cuticle damage (Figure 1F–I; Table 1). In contrast, only a small fraction of unmated hermaphrodites showed such damage (Figure 1C–E; Table 1). Thus, continuous mating conditions can damage the cuticle surface of the vulva.

Since QG2288 males induced a higher fraction of damaged vulvae than did DR461 (*him-5*) males, the area of cuticle damage induced by these males was also measured. However, the extent of cuticle damage was not statistically different (Mann-Whitney U p-value = 0.12) between the males of strains DR461 (mean area of cuticle damage = 1732 µm^2^; range = 620–3452 µm^2^) and QG2288 (mean area of cuticle damage = 647 µm^2^; range = 219–1140 µm^2^).

**Figure 2:**
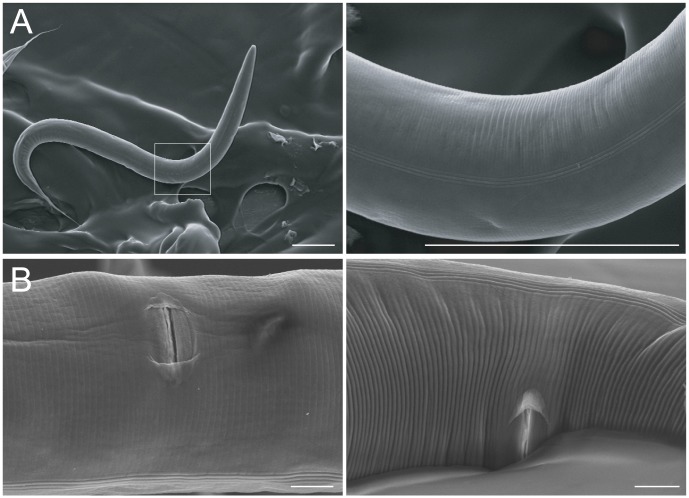
Soil conditions do not cause cuticle damage. SEM images of hermaphrodites grown in soil plate conditions. (A) Low- (left) and high-magnification (right) images of a worm grown for five days in soil, showing pristine ultrastructure of the cuticle. The three parallel alae (ridges running transversely across the cuticle) are clearly visible in the high-magnification view. (B) Soil conditions also do not damage cuticle surrounding the vulva. Two representative specimens are shown. Scales bars represent 100 microns in A and 10 microns in B.

The marked increase in cuticle damage due to mating led us to examine whether other stresses may also promote such damage. Laboratory conditions are quite different from the natural ecological context of *C*. *elegans* [31]. *Caenorhabditis* is thought to proliferate on rotting fruit adjacent to soil [32], so we examined worms cultured in a bacteria-soil mixture atop standard agar medium for five days (see Methods). However, hermaphrodites on these “dirt plates” were not obviously abraded (Figure 2A, n = 12), and showed no discernable difference in overall cuticle morphology from hermaphrodites grown on standard media (data not shown). Moreover, soil-cultured hermaphrodites lacked any apparent cuticle damage around the vulva (Figure 2B; n = 7), suggesting that the vulva is not particularly susceptible to such damage. Thus, mating may be particularly harmful for hermaphrodites, as traversing through potentially harsh conditions appears to not promote such physical damage.

## Discussion

We have used SEM to demonstrate that prolonged mating is associated with vulval cuticle damage in *C*. *elegans* hermaphrodites. A previous study [20] utilized a number of genetic experiments to hypothesize that the lifespan cost to mating in *C. elegans* is due to the act of mating itself, and not due to any metabolic cost due to increased progeny production. Particularly, the use of sperm-defective and spermless males still induced a lifespan reduction in hermaphrodites that was comparable to that induced by wild-type males [20]. Thus, it was concluded that physical damage or infection due to the physical act of mating itself was likely the cause of the mating-dependent lifespan reduction [20]. However, this hypothesis was not interrogated by direct microscopic observation. Our results are consistent with these previous predictions. However, it remains unclear whether the observed cuticular damage contributes to reduced longevity.

The physical damage hypothesis was supported by the ability of mutant males that cannot transfer sperm to reduce hermaphrodite lifespan [20]. The possibility therefore remained that hermaphrodites were harmed by male seminal fluid. Indeed, seminal fluid is responsible for mating-induced harm of females in a number of species [33]. Furthermore, two recent studies [22], [23] provided evidence that the major causes of mating-induced harm in *C. elegans* hermaphrodites are seminal fluid and diffusible pheromone-like molecules. Strikingly, a number of mutations (in the genes *ins-11*, *che-13*, *gon-2*) render hermaphrodites resistant to mating-induced death [22], [23]. This suggests that the hermaphrodite gonad and signaling pathway components are necessary for mating-induced death. Additionally, mating induces changes in hermaphrodite cuticular gene expression [18] and body size shrinkage [23] in hermaphrodites. Cuticular damage may therefore reflect mating-induced changes in hermaphrodite physiology, indicative of the early stages of mating-induced shrinkage. Furthermore, differences in the extent of cuticle damage between N2 and QG2288 may be due to differences in genetic background affecting hermaphrodite cuticle strength. While visually striking, we cannot exclude the possibility that cuticle damage resulting from mating is of little consequence for hermaphrodite lifespan.

The cuticle is a critical organ for nematode survival, as it is the exoskeleton and confers environmental protection [34]. Presumably an intact cuticle is more effective than a damaged one, at least under some circumstances. Although the extent to which mating-induced cuticle damage impairs cuticle function is uncertain, it is notable that another potentially stressful condition (growing the animals on soil plates) did not damage the cuticle (Fig. 2). Thus, crawling through soil appears to impact cuticle surface morphology far less than does copulation.

Mating-induced physical damage to females has been observed in a number of non-nematode animals [16]. Recovery of giant squid revealed spermatophores embedded in female arms is suggestive of mating-induced physical damage [35]. In bedbugs, reproduction involves “traumatic insemination,” wherein during copulation the male intromittent organ pierces the female abdominal wall, which is costly for females [36]. Additionally, mating promotes genital damage in bean weevils that reduces female lifespan [13]. Such mating-induced female harm is oftentimes framed within the context of sexually antagonistic coevolution [16], in which harm to females is a consequence of strategies that increase male fitness. This is distinct from reproductive costs that may be related to metabolic tradeoffs between reproduction and somatic maintenance [20], [22], [23], another long-standing explanation for lifespan costs to mating [1].
